# Evidence Against the Causal Relationship Between a Putative Cis-Regulatory Variant of *MYH3* and Intramuscular Fat Content in Pigs

**DOI:** 10.3389/fvets.2021.672852

**Published:** 2021-06-02

**Authors:** Cong Huang, Liepeng Zhong, Xiaoxiao Zou, Yizhong Huang, Liping Cai, Junwu Ma

**Affiliations:** State Key Laboratory for Swine Genetics, Breeding, and Production Technology, Jiangxi Agricultural University, Nanchang, China

**Keywords:** *MYH3*, 6-bp deletion variant, causal mutation, meat quality traits, intramuscular fat content

## Abstract

Improving meat quality has become the main goal of modern pig breeding. Intramuscular fat content (IMF) is an important trait influencing meat quality of livestock, but the molecular mechanism behind this trait is still unclear. Recently, Cho et al. reported the discovery of the first causal mutation affecting IMF and red flesh color (a*) in pigs, namely *XM_013981330.2:g*.−*1805_*−*1810del*, a 6-bp deletion variant in the porcine *MYH3* promoter region. The objective of this study was to reassess the causality of this mutation for its potential commercial application. By Sanger sequencing, we firstly identified several new variants (including a 4-bp deletion) at or near the 6-bp deletion site, which formed four haplotypes in multiple breeds. Unexpectedly, the 6-bp deletion allele, previously determined as the *MYH3 Q* allele because of its significantly positive effect on IMF and a*, was found not only in Chinese indigenous breeds, but also in four western commercial breeds with relatively lower IMF levels, including Duroc, Large White, Landrace and Pietrain. More surprisingly, we found that the *MYH3 Q* allele and the haplotypes harboring it had no significant effects on IMF, marbling and color score in three large-scale divergent pig populations: the heterogeneous F6 and F7 pigs and commercial crossbred Duroc × (Landrace × Yorkshire) pigs. Transient transfection analysis in porcine satellite cells showed that the 6-bp deletion variants had a negligible effect on transcription of reporter gene, but could attenuate the MRF (myogenesis regulatory factors)-induced increase in luciferase activity of the *MYH3* promoter vector. The *MYH3* protein level in muscle did not differ significantly among the haplotype groups. Therefore, our results cannot support the causal relationship between the 6-bp deletion in *MYH3* and IMF trait, suggesting that the causal mutation for the IMF QTL on SSC12 needs to be further identified.

## Introduction

Intramuscular fat (IMF) content plays an important role in determining the eating quality of pork, such as tenderness, juiciness and flavor. Therefore, the pork with high IMF content and good eating quality is generally favored by consumers (1, 2). There are considerable variations in IMF contents among different pig breeds, especially between western commercial pig breeds and Chinese native pig breeds (3–5), which suggests that heredity is a major determinant of IMF. In fact, a large number of quantitative trait loci (QTL) or candidate genes for IMF have been identified by linkage and genome-wide association (GWA) studies. However, despite the progress achieved in this field in recent years, molecular mechanisms behind IMF trait remains elusive, as few causative genes or causative mutations affecting IMF content of pork have been determined (6–10).

Recently, a study conducted by Cho et al. presented evidences that a 6-bp deletion (*XM_013981330.2:g*.−*1805_*−*1810del*) in the promoter region of *MYH3* is the first causal mutation that was identified for IMF and red flesh color (a*) in domestic pigs (11). They firstly identified a 488.1-kb critical region on porcine chromosome 12 (SSC12) that affects both IMF and a* by a joint linkage-linkage disequilibrium analysis in two independent F2 crosses between Korean native pigs (KNPs) and Western commercial breeds (Landrace and Duroc). In this critical region, only the *MYH3* gene, encoding myosin heavy chain 3, was found to be preferentially overexpressed in the skeletal muscle of KNPs than in that of Western commercial breeds. They further validated *MYH3* as a quantitative trait gene (QTG) using transgenic mice, and then discovered the *XM_013981330.2:g*.−*1805_*−*1810del* variant in the 5′-flanking region of *MYH3*, which deletion (*Q*) allele carriers exhibited significantly higher values of IMF and a* than wild-type (*q*) allele carriers. They demonstrated that this 6-bp deletion variant could abrogate the binding of the regulatory myogenic regulatory factors (MRFs, i.e., *MYOD, MYOD, MYF5* and *MRF4*) and act as a significantly weaker repressor, resulting in increased expression of *MYH3* in the skeletal muscle. In addition, they found that the *MYH3 Q* allele occurred exclusively in Asian domestic breeds (such as Chinese Neijiang, Chinese Putian, Chinese Xiang, and KNP) and Asian wild boars, but was almost absent in European and African wild boars, as well as European commercial pig breeds (including Large White, Landrace and Duroc, etc.), which indicates that this allele is of Asian origin.

In general, the effects of a causal mutation can be replicated in different populations. Unfortunately, we herein present genetic and functional evidence against the causal relationship of the *XM_013981330.2:g*.−*1805_*−*1810del* with meat quality traits. We found that the *MYH3 Q* allele was present not only in Chinese native breeds but also in four western commercial breeds including Duroc, Large White, Landrace and Pietrain. The effects of the 6-bp deletion variant and a novel 4-bp deletion variant at the same locus identified by us on the meat quality traits including IMF, marbling and color score were not significant in either heterogenous stock F6 pig population or a commercial hybrid Duroc × Landrace × Yorkshire (DLY) pig populations. And these two deletion variants were also not significantly associated with a* in both the heterogeneous F7 pigs and the DLY pigs. The protein expression level of *MYH3* in porcine longissimus muscle did not differ significantly among animals with different genotypes or haplotype combinations at the target *MYH3* locus. The findings thus demonstrate that the *MYH3 XM_013981330.2:g*.−*1805_*−*1810del* is unlikely to be causal mutation for the meat quality traits.

## Materials and Methods

### Animals and Phenotypes

A total of 1391 pigs, including 751 F6 pigs and 587 F7 pigs from a heterogenous stock and 546 Duroc × (Yorkshire × Landrace) (DYL) pigs were used in this study. The pig heterogenous stock was established from 8 founder divergent pig breeds including 4 Chinese indigenous breeds (Erhualian, Laiwu, Bamaxing, and Tibetan) and 4 Western commercial breeds (Duroc, Large White, Landrace, and Pietrain), which has been described in greater detail elsewhere (12). The DYL pigs were all raised on a farm of the Jiangxi Guohong Group Co. Ltd. in Jiujiang city (Jiangxi Province), and they were randomly selected from the offspring of about 20 adult Duroc boars and hundreds of L × Y sows on the farm. All heterogenous animals were slaughtered at the age of 240 ± 3 days, and the DYL pigs were slaughtered at 180 ± 3 days. The total number of slaughter batches of F6, F7 and DYL were 23, 19, and 17, respectively. About 30–36 pigs were slaughtered in each batch. Meat quality measurement was conducted as reported previously (13). Briefly, the *longissimus dorsi* (LD) were collected from the left side of each carcass between the 11th-rib and the first lumbar vertebra for measurement of meat quality traits. Meat redness (a*) was measured by CM-2600d/2500d Minolta Chroma meter. Meat color scores (ranging from 1 to 6, with 1 = pale and 6 = dark), marbling scores (ranging from 1 to 10 with 1 = devoid and 10 = overly abundant) were subjectively evaluated according to National Pork Producer Council (NPPC) guidelines (14) and the IMF content was determined by using the Soxhlet method (15). Except for the IMF of the F7 pigs, all phenotypic data of the three populations have been collected.

### Genotyping and Sequencing of the *MYH3* Locus

Genomic DNA was isolated from the ear tissue of each pig using a standard phenol/chloroform method and dissolved in Tris-EDTA buffer. The DNA quality and concentration were determined using a Nanodrop-1000 spectrophotometer (Thermo Fisher, USA). DNA samples from 94 F7 pigs were genotyped for the 6-bp deletion variant, *XM_013981330.2:g*.−*1805_*−*1810del*, in the promoter of *MYH3* using the PCR-RFLP method developed previously (11). A fragment with the expected size of 457bp covering the *XM_013981330.2:g*.−*1805_*−*1810del* mutation was amplified by PCR (primers: forward 5′ – GTG GGC AAA GGG ATG AAG - 3′; reverse 5′ – GGA ATA AGA ATG GGC AAA CG - 3′). And the amplicons were digested with the restriction endonuclease, *Hpy*CH4IV. Sanger sequencing of the PCR products from these F7 animals were conducted to determine the sequence variations located at and near the 6-bp deletion, and a set of haplotypes were subsequently constructed with the adjacent variations we identified. In addition, the Sanger sequencing method was also applied to determine the distribution of haplotypes in 51 pigs from 8 founder breeds of the heterozygous stock and in 546 DYL pigs. For all the F6 and F7 individuals, their haplotypes in the target area could be inferred from the next-generation sequencing (NGS) data (Supplementary Table 1), which were generated using the Illumina Xten platform (Illumina Inc. San Diego, CA). We performed whole-genome NGS for each founder animals (F0) with an average coverage depth ~30 ×. The genome of each F6 and F7 animal was sequenced to 7.8-fold average coverage. The methods for read mapping and genotype calling in our NGS data was described previously (12).

### Dual-Luciferase Assay and Western Blotting Analysis

Pig muscle satellite cells (MZ-3319, mingzhoubio) were cultured in DMEM (Hyclone) containing 10% FBS (Gibco) and 1% penicillin-streptomycin reagent (Solarbio). Cells were grown at 37°C in humidified air containing 5% CO_2_ (Thermo) until transfection. Then 20 μl lipo2000 were dissolved in 980 μl Opti-mem serum-free medium followed by mixture and setting for 5 min at room temperature. All *MYH3* luciferase reporter constructs were generated by subcloning 4 different haplotype sequences of about 90 bp centered by *XM_013981330.2:g*.−*1805_*−*1810del* in front of the luciferase gene in the pGL3 basic vector. The MZ-3319 cells were seeded by adding 1 ml cell suspension containing 5 × 10^5^ cells/ml to each well of 6-well culture plate and then placed in a cell incubator at 37°C in humidified air containing 5% CO_2_ until cells grow to 70–80% degrees of fusion. The mixture of 5 μl synthetic sequences and 245 μl Opti-mem of five synthetic sequences were added to the 200 μl mixtures of 4 μl lipo2000 and 196 μl Opti-mem serum-free medium. All transfection solutions static at room temperature for 20 min. Before transfection, the cells were rinsed once with sterile PBS and then 2 ml serum-free medium was added to each well of the cell plates. All transfection solutions were added slowly to the serum-free medium of each cell and then put the plates in cell incubator for 6 h at 37°C. After removing serum-free medium, complete medium was added to culture the cells. Cells were collected to carry out the subsequent experiments after 48 h transfection. Transfected cells were rinsed in PBS and then lysed in 1 × passive lysis buffer. Luciferase assays were performed using a Dual-Luciferase Reporter Assay System (Promega) to measure Renilla (internal control) and firefly (reporter construct) luciferase values. MRF genes, including *MYF5, MYOD, MYOG*, and *MRF4*, were co-transfected with an *MYH3* promoter and an internal control vector into MZ-3319 cells. After 48 h, the effects of four MRF gene on *MYH3* promoter activity were assayed using the Dual-Luciferase Reporter Assay System.

Western blot analysis was used for measuring the protein expression of the *MYH3* gene in *longissimus dorsi* muscle of 18 F7 pigs with different genotypes of the *MYH3* locus. In brief, total protein was isolated from cells using RIPA buffer (R0020; Solarbio) containing a PMSF protein inhibitor (100 mM; 1.5 ml). Protein concentrations were quantified by a BCA Protein Assay Kit (PICPI23223, Thermo Fisher Scientific, Inc., USA) after muscle tissue was totally lysed followed by centrifugation for 15 min at 4°C, 12,000 g. Proteins were separated by SDS-PAGE after the mixture of protein and SDS-PAGE loading buffer was kept in boiling water for 10 min (Leica HI1210, Leica Biosystems). Then the protein was semi-dry transferred onto immobilon-NC transfer membranes (HATF00010, Millipore Corporation), blocked in 5% fat-free milk for 1 h at room temperature and then incubated with primary antibodies (ab124205, Abcam plc.) in 5% milk overnight at 4°C. After being washed three times by TBST, the membrane was then incubated with secondary antibody for 1 h at 37°C. The bands of target proteins were visualized through an ECL imaging system (Tanon 5200, Shanghai).

### Statistical Analysis

Association analyses between the *MYH3* polymorphisms (including *XM_013981330.2:g*.−*1805_*−*1810del*) and target traits in each population were conducted by using a general linear model (GLM) in R software (version 3.6.2). The linear model is shown below:

(1) $$\begin{array}{r} Y_{ijk}\;=\;\mu +S_{i}+B_{j}+G_{k}+\;W_{ijk}+\;e_{ijk} \end{array}$$

where, *Y*_*ijk*_ is the phenotypic value of each trait, μ is overall mean for each trait, *S*_*i*_ is the effect of gender, *B*_*j*_ is the effect of slaughter batch, *G*_*k*_ is the effect of the *MYH3* genotypes or haplotype combinations, *W*_*ijk*_ is the covariate of slaughter weight, and *e*_*ijk*_ is the random error.

The least square mean ± standard error for each haplotype combination was obtained using the *lsmeans()* function in the lsmeans R package. Tukey pair-wise comparisons were subsequently conducted to get difference between groups by using the *cld()* function in the multcomp *R* package. The *P*-value < 0.05 was considered as significant.

## Results

### Discovery of New Variants and Haplotypes in the Promoter of *MYH3*

Considering that the heterogenous F7 pigs had genetic polymorphisms of eight breeds, we conducted fragment analysis on 94 F7 pigs to uncover mutations around the 6-bp deletion, *XM_013981330.2:g*.−*1805_*−*1810del*, in the promoter of *MYH3* by PCR-based Sanger sequencing and PCR-RFLP methods. Sanger sequencing showed four haplotypes and seven haplotype combinations in those pigs (Table 1 and Supplementary Figure 1). Two of the haplotypes (*H1* and *H2*) are previously known and the other two (*H3* and *H4*) are new. The haplotypes consisted of a single nucleotide insertion site (insert T or C), a SNP site (G > A) just 1-nt upstream of the 6-bp deletion and a deletion site (6-bp or 4-bp deletions). However, we noted that the PCR-RFLP with *Hpy*CH4IV endonuclease can only distinguish the mutate type *H2* from the other three haplotypes, while the two novel haplotypes *H3* and *H4*, albeit harboring the 6-bp deletion (the *MYH3 Q* allele) and the 4-bp deletion, respectively, were wrongly judged as corresponding to the *q* allele as the wild-type haplotype *H1* (Table 1). Although the *H2* and *H3* differ by only one SNP allele, the DNA strand with *H3* cannot be cleaved by *Hpy*CH4IV enzyme. Therefore, the PCR-RFLP method with *Hpy*CH4IV is not suitable for genotyping of the *MYH3 XM_013981330.2:g*.−*1805_*−*1810del* in pig populations with multiple haplotypes.

**Table 1:**
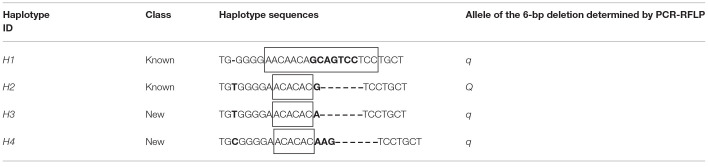
Identification of haplotypes containing the *MYH3* 6-bp deletion variant in heterogenous F7 pigs by Sanger sequencing.

*PCR-RFLP, PCR amplification and subsequent HpyCH4IV digest*.

*Different nucleotide repeats in the haplotypes are boxed*.

### The Distribution of the Four Haplotypes in Eight Breeds

To detect the origin and distribution of the four haplotypes in eight founder breeds of the heterogenous stock pig population, we performed PCR and Sanger sequencing on genomic DNA from the F0 pigs in the population. Table 2 shows that the *H1* haplotype was present in all the breeds. Surprisingly, the previously recognized *H2* haplotype harboring the *MYH3 Q* allele was found not only in the three Chinese native breeds (Tibetan, Erhualian and Laiwu) except Bamaxiang, but also in the four western commercial pig breeds, including Duroc, Large White, Pietrain and Landrace (Table 2). The new haplotype *H3*, also harboring the *MYH3 Q* allele, was detected exclusively in Bamaxiang, while another new haplotype *H4* distributed in Chinese Laiwu breed and the four commercial pig breeds. So the data demonstrates that all the 8 breeds had carriers of the *MYH3 XM_013981330.2:g*.−*1805_*−*1810del* variant which was considered to be responsible for the observed SSC12 QTL effect on IMF and a*.

**Table 2:** The haplotype combinations found in 8 founder breeds of the heterogeneous stock population.

**Breeds**	**Haplotype combinations**						
	***N***	**11**	**12**	**13**	**14**	**22**	**33**
Chinese Bamaxiang	5			2			3
Chinese Tibetan	7	4	2			1	
Chinese Erhualian	6		5			1	
Chinese Laiwu	6		2		2	2	
Duroc	8	3	2		3		
Large White	6	3	2		1		
Pietrain	6	5	1				
Landrace	7	4	2		1		

*N: The total number of animals in each pig breed*.

### The Effect of the Haplotypes on Meat Quality Traits in Three Pig Populations

Next, we sought to evaluate the effect of the four haplotypes on 4 meat quality traits including a*, color score, marbling score and IMF, in 751 F6 and 587 F7 pigs whose haplotypes were obtained from their next-generation sequencing (NGS) data. Based on the Sanger sequencing results of 94 F7 samples, the NGS-derived haplotypes were confirmed to be correct. Seven haplotype combinations were identified in both the F6 and F7 pigs. In each of the two populations, the number of animals with the 11, 12 haplotype combinations exceeded 133, while the number of samples with the 22 haplotype combination (having two copies of the 6-bp deletion) was only 29 and 13 in F6 and F7, respectively (Table 3). The result of association analysis in F6 pigs showed that the haplotypes was significantly (*P* < 0.05) associated with a* and IMF, but not color score and marbling (Table 3). Particularly, the difference in a* among the 7 haplotype combinations was highly significant (*P* = 3.06E-07), and the average of a* values were significantly lower in the pigs with the 11 and 13 haplotype combinations than those with 12 and 14 haplotype combinations. The means of IMF for the 11, 12, 13, and 14 haplotype combinations did not differ from each other. The difference in the IMF trait was only found between the 23 and 24 haplotype combinations in the F6 pigs. In contrast, the haplotypes was not significantly associated with a* in the F7. Although the 12 and 14 haplotype combinations from the F7 differed significantly (*P* < 0.05) in color score and Marbling, there was no significant difference in the two traits between the 11, 12, and 22 haplotype combinations in this population. The results suggest that neither haplotypes nor variants in the haplotype combinations including the *XM_013981330.2:g*.−*1805_*−*1810del* could significantly and positively influence both a* and IMF (or marbling).

**Table 3:** Comparison of four meat quality traits among the haplotype combinations in the heterogenous F6, F7, and DYL pig populations (least square mean ± standard error).

**Traits**	**Haplotype combinations**							***P-*value**
	**11**	**12**	**13**	**14**	**22**	**23**	**24**	
F6 (*N* = 751)								
*N*	272	224	114	58	29	34	20	
a*	0.99 ± 0.08^a^	1.54 ± 0.09^c^	1.08 ± 0.12^ab^	1.74 ± 0.16^c^	1.73 ± 0.23^bc^	1.60 ± 0.21^abc^	1.70 ± 0.28^abc^	3.06E-07
Color score	2.74 ± 0.04^a^	2.83 ± 0.05^a^	2.73 ± 0.06^a^	2.73 ± 0.09^a^	2.91 ± 0.13^a^	2.79 ± 0.11^a^	2.59 ± 0.15^a^	0.495
Marbling	2.64 ± 0.06^a^	2.80 ± 0.07^a^	2.85 ± 0.10^a^	2.57 ± 0.14^a^	3.10 ± 0.19^a^	2.88 ± 0.17^a^	2.38 ± 0.23^a^	0.053
IMF (%)	2.08 ± 0.04^ab^	2.17 ± 0.05^ab^	2.08 ± 0.07^ab^	1.98 ± 0.1^ab^	2.21 ± 0.13^ab^	2.37 ± 0.12^b^	1.68 ± 0.16^a^	0.011
F7 (*N* = 587)								
*N*	227	133	70	91	13	26	27	
a*	1.07 ± 0.11^a^	1.49 ± 0.15^a^	0.86 ± 0.20^a^	1.07 ± 0.17^a^	1.53 ± 0.45^a^	1.20 ± 0.32^a^	0.99 ± 0.32^a^	0.146
Color score	3.09 ± 0.04^ab^	3.19 ± 0.06^b^	2.91 ± 0.08^ab^	2.86 ± 0.07^a^	2.88 ± 0.18^ab^	3.07 ± 0.13^ab^	3.18 ± 0.13^ab^	0.003
Marbling	3.22 ± 0.06^ab^	3.37 ± 0.09^b^	3.04 ± 0.11^ab^	2.93 ± 0.10^a^	2.92 ± 0.26^ab^	3.24 ± 0.18^ab^	3.33 ± 0.18^ab^	0.015
DYL (*N* = 546)								
*N*	434	73	5	33	NA	1	NA	
a*	1.20 ± 0.06^a^	1.12 ± 0.11^a^	1.24 ± 0.41^a^	1.25 ± 0.17^a^	NA	0.76	NA	0.660
Color score	3.02 ± 0.03^a^	3.05 ± 0.06^a^	2.89 ± 0.23^a^	3.06 ± 0.09^a^	NA	3	NA	0.868
Marbling	2.72 ± 0.04^a^	2.69 ± 0.07^a^	2.53 ± 0.27^a^	2.75 ± 0.11^a^	NA	2	NA	0.661
IMF (%)	1.82 ± 0.03^a^	1.80 ± 0.07^a^	1.70 ± 0.25^a^	1.94 ± 0.10^a^	NA	0.73	NA	0.389

*Means with the same superscript letter (a-c) in a row are not significantly (P < 0.05) different*.

*IMF, intramuscular fat*.

*DYL, Duroc × (Landrace × Yorkshire) commercial hybrid pig*.

Given that the four haplotypes were also found in Duroc, Landrace and Large White (or Yorkshire), we then examined whether the four haplotypes were associated with the meat quality traits in their hybrid DLY. In 546 DLY pigs, 5 haplotypes combinations including 11, 12, 13, 14, and 23 were detected through Sanger sequencing. The most predominant haplotype combination was 11, followed by 12 and 14, whose frequencies were 74.5, 13.4, and 6.0%, respectively (Table 3). However, we demonstrated that there was no significant difference in the analyzed 4 meat quality traits (a*, color score, marbling, and IMF) among those haplotype combinations (Table 3). Thus, the results of the analysis in the two populations strongly question that *XM_013981330.2:g*.−*1805_*−*1810del* is a causal mutation of the meat quality traits.

### Functional Implication of the Haplotypes on *in vitro* and *in vivo* Gene Expression

**Figure 1:**
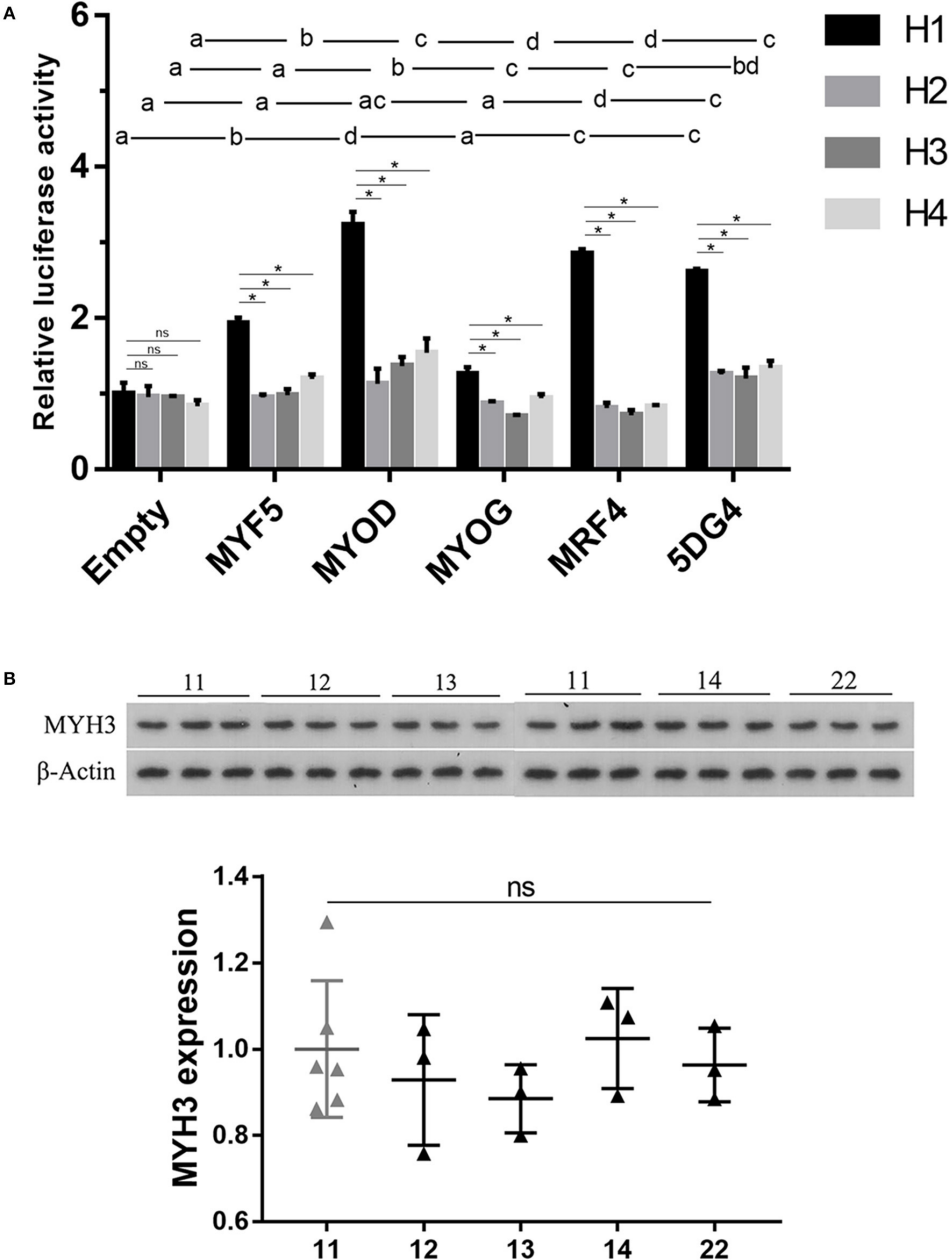
Analysis of the effect of the identified four haplotypes on gene expression analyses of (upper) and western blotting assays of 18 LD muscle samples (lower). **(A)** Transient co-transfection assays of porcine muscle satellite cells. The various haplotype-luciferase reporter constructs were co-transfected with either one of the four MRF constructs or the four MRFs (5DG4). The luciferase activity did not differ significantly between groups with the same letter. Data histograms and error bars represent the mean ± standard error of triplicate independent samples. ns: *P* > 0.05, *P < 0.05. **(B)** Western blot analysis of *MYH3* protein levels in muscle from animals with different haplotype combinations. The gene expression level was normalized from the gray value calculated by ImageJ. Data are presented as mean ± SD. ns: *P* > 0.05, *P < 0.05.

The above-mentioned results motivated us to investigate whether the four haplotypes at 5′-flanking region of *MYH3* affect gene expression. To this end, we compared the four haplotype (*H1, H2, H3*, and *H4*) promoter driven luciferase activities through transient transfection assays in porcine satellite cells. The cells were co-transfected with reporter constructs containing one of the four haplotypes and an empty expression vector (as a control), each of the four MRFs (*MYH5, MYOD, MYOG*, and *MRF4*) expression vectors or all the four MRFs expression vectors. No significant differences were observed in luciferase activities among the four haplotypes in control cells transfected with empty expression vector (Figure 1A). Notably, the wild-type haplotype *H1* promoter construct exhibited a nearly 2–3-folds increase (*P* < 0.05) in luciferase activities after expression of either one or all of the MRFs. In contrast, these MRFs did not always enhance gene expression in the three mutant haplotypes constructs, and the extent to which they altered luciferase activities was significantly reduced (Figure 1A). The results suggest that the wild-type haplotype may be serve as an enhancer activated by the MRFs, but the mutations on it could impair the activation of transcription factor and the function of the element.

To further assess the effect of the four haplotypes on *MYH3* gene expression, we performed western blotting analysis on the protein extracted from the *longissimus dorsi* muscle of 18 F7 pigs with five different haplotype combinations (11, 12, 13, 14, and 22; 3 samples per haplotype combination). We did not observe significant difference in the *MYH3* protein levels between any pair of the haplotype combinations (Figure 1B), suggesting that the mutations detected in the *MYH3* promoter had negligible effect on *MYH3* expression in adult muscle.

## Discussion

Identification of major genes affecting meat quality traits will help to improve meat quality efficiently and yield significant economic benefits. This is clearly reflected in the breeding application of several causal genes and their causal mutations, such as *RYR1* R615C*, PRKAG3* R200Q, and *IGF2* intron3-g.3072G>A (16–18). The causalities between these genes and several meat quality traits have been endorsed by multiple studies. So far, only one study has shown that the 6-bp deletion, *XM_013981330.2:g*.−*1805_*−*1810del*, in the promoter of *MYH3* is very likely the first identified causal mutation underlying IMF. Its researchers also demonstrated that the significant effects of this mutation on IMF and a* were replicated in two independent cohorts: a Landrace × KNP F_2_ intercross and a Duroc × KNP F_2_ intercross. However, due to that the so-called *MYH3 Q* allele (i.e., 6-bp deletion allele) favorably associated with the meat quality traits in the two F2 populations originated only from the KNP pigs and not from other founder breeds, the length of linkage disequilibrium block surrounding the mutation would be too long (>700 kb) to make it difficult to determine whether the 6-bp deletion variant is the causal mutation itself or a marker closely linked to the causal mutation. Therefore, examining the segregation of the 6-bp deletion and its effect on meat quality in pigs with other genetic backgrounds may help verify whether or not it is a causal mutation. In this study, we first determined that *XM_013981330.2:g*.−*1805_*−*1810del* was segregating in the heterogeneous F6 and F7 pig populations derived from the intercross between 8 divergent founder breeds. But surprisingly, we further found that this mutation occurred in all the 8 founder breeds, including 4 Chinese indigenous breeds (Bamaxiang, Laiwu, Erhualian, and Tibetan) and 4 western commercial breeds (Duroc, Landrace, Large White and Pietrain). The result is not in agreement with the findings of Cho et al. (11) who did not find the *MYH3 Q* allele in Western commercial breeds. Therefore, their inference that the *MYH3 Q* allele is of Asian origin and likely predates domestication needs to be re-examined.

We further showed that the *MYH3 Q* allele had no significant impact on IMF, marbling and color score in the F6 or F7 populations, which is contrary to the result of the previous study (11). It is worth mentioning that the genome-wide association (GWA) study in the F6 pigs have identified a QTL for both IMF (*P*-value = 1.31E-22) and a* (*P*-value = 1.64E-26) located close to the *MYH3* gene on SSC12 (data not shown). Therefore, the 6-bp deletion variant could not be the cause of the IMF QTL on SSC12 detected in the F6 population. In addition, the *MYH3 Q* allele was also present in 78 (14.2%) out of the 546 DLY pigs tested, while our previous GWAS study showed that there was no QTL for IMF or a* adjacent to the *MYH3* gene in this DLY population (13). Obviously, the effects of the *MYH3 Q* allele on the 4 meat quality traits were also negligible in the DLY pigs. Thus, we replicated the results in both the F6 and DLY populations with and without the SSC12 QTL, respectively, strongly suggesting that the *XM_013981330.2:g*.−*1805_*−*1810del* is not the causal mutation for the meat quality traits.

It was noted that the haplotypes H2, H3, and H4 contains the same (AC)_3_ nucleotide repeats, whereas the H1 has (AAC)_2_ (CAG)_2_ (TCC)_2_ (there is a C that needs to be used for both the first 2 trinucleotide repeats) (Table 1). Since microsatellite segments may help open DNA structure and even cause disease (19), we evaluated whether this kind of variation was more likely to affect the meat quality traits. The association analysis showed that the nucleotide repeats had negligible effect on the four meat quality traits (Supplementary Table 2).

The transient transfection analysis of the porcine fibroblast cells performed by Cho et al. showed that the *MYH3 q* variant acts as a repressor element, whereas the *MYH3 Q* variant functions as a significantly weaker repressor, leading to increased transcription of *MYH3* in skeletal muscle of KNPs or *Q* allele carriers. However, we did not confirm that the 6-bp deletion variant can cause a significant change in the *MYH3* protein level in porcine skeletal muscle and the transcriptional activities of the luciferase reporter constructs containing the *MYH3 Q* and *q* haplotypes. Interestingly, we found that the *MYH3 q* variant site may be triggered to become an enhancer by overexpression of one of the MRFs in porcine muscle satellite cells, but the *MYH3 Q* variant significantly weakened the stimulatory effect of the MRFs. Some studies showed that Western lean-type pig breeds (such as Landrace, Large White, Yorkshire) had a significantly higher expression level of *MYH3* than Chinese indigenous fat-type pig breeds (such as Tongcheng, Mashen, Laiwu) (20–22). While, the expression level of *MYH3* gene was higher in Chinese Lantang pigs than in Landrace at the fetal stage (23). The regulatory mechanism of the variation in the expression of *MYH3* in muscle throughout swine lifespan remains to be elucidated.

## Conclusions

Our results show that the *XM_013981330.2:g*.−*1805_*−*1810del* in the promoter of porcine *MYH3* did not significantly affect the meat quality traits in both Chinese × European crossbred pigs and Western commercial DLY crossbred pigs, which is not consistent with the assertion that this variant is the causal mutation underlying the meat-quality related QTL on SSC12. The *MYH3* gene is still one of the important candidate genes for the QTL. The molecular mechanism of the SSC12 QTL needs further study.

## Data Availability Statement

The data generated for the study are deposited in the (National Center for Biotechnology Information GenBank, https://www.ncbi.nlm.nih.gov/genbank/) repository, accession number (MW769011–MW769701).

## Ethics Statement

The animal study was reviewed and approved by The Ethics Committee of Jiangxi Agricultural University.

## Author Contributions

CH and LZ contributed to the experiments, data analysis, and original draft writing. XZ conducted cell culture and experiments. LC and JM played leading roles in article revision, supervision, funding acquisition while YH assisted collecting the samples required for data analysis and experiments. All authors read and approved the submitted version.

## Conflict of Interest

The authors declare that the research was conducted in the absence of any commercial or financial relationships that could be construed as a potential conflict of interest.

## Supplementary Material

Supplementary Figure 1 Discovery of four haplotypes and 8 haplotype combinations at the *MYH3 XM_013981330.2:g*.−*1805_*−*1810del* promoter region by Sanger sequencing.

Supplementary Table 1 The corresponding relationship between four haplotypes identified by Sanger sequencing and the alleles of the 5 variants detected by the next-generation sequencing (NGS).

Supplementary Table 2 Comparison of four meat quality traits between genotypes of nucleotide repeats in the heterogenous F6, F7, and DYL pig populations.
