# Supplementary figures and images for: Evidence Against the Causal Relationship Between a Putative Cis-Regulatory Variant of MYH3 and Intramuscular Fat Content in Pigs
Source: Front Vet Sci. 2021 Jun 2;8:672852. doi: 10.3389/fvets.2021.672852 (PMC8206472; doi:10.3389/fvets.2021.672852)

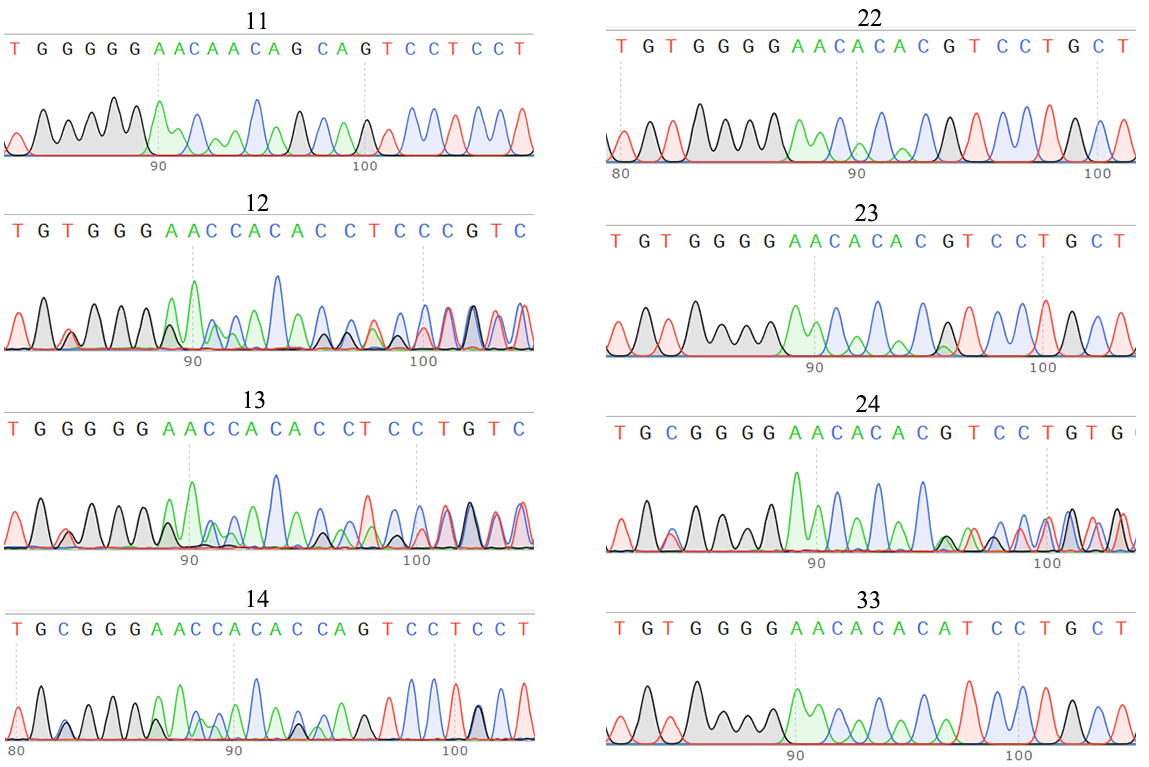

Supplement: Supplementary Figure 1 — Discovery of four haplotypes and 8 haplotype combinations at the MYH3 XM_013981330.2:g.−1805_−1810del promoter region by Sanger sequencing. [file Image_1.TIF]
