# Supplementary material for: Evidence Against the Causal Relationship Between a Putative Cis-Regulatory Variant of MYH3 and Intramuscular Fat Content in Pigs
Source: Front Vet Sci. 2021 Jun 2;8:672852. doi: 10.3389/fvets.2021.672852 (PMC8206472; doi:10.3389/fvets.2021.672852)
Supplement: Supplementary Table 2 — Comparison of four meat quality traits between genotypes of nucleotide repeats in the heterogenous F6, F7, and DYL pig populations. [file Table_2.DOCX]

**TABLE S2 | Comparison of four meat quality traits between genotypes of nucleotide repeats in the heterogenous F6, F7, and DYL pig populations.**

| **Traits** | **Genotype of nucleotide repeats** | | | ***P-*value** |
| --- | --- | --- | --- | --- |
|  | **zz** | **Zz** | **ZZ** |  |
| **F6 (N = 751)** |  |  |  |  |
| **N** | **272** | **396** | **83** |  |
| a^*^ | 0.99 ± 0.08^b^ | 1.43 ± 0.06^b^ | 1.68 ± 0.14^a^ | 1.49E-06 |
| Colorscore | 2.74 ± 0.04^a^ | 2.79 ± 0.03^a^ | 2.78 ± 0.07^a^ | 0.73 |
| Marbling | 2.64 ± 0.06^a^ | 2.78 ± 0.05^a^ | 2.83 ± 0.11^a^ | 0.14 |
| IMF (%) | 2.08±0.04^a^ | 2.11±0.04^a^ | 2.14±0.08^a^ | 0.69 |
| **F7 (N = 587)** |  |  |  |  |
| **N** | **227** | **294** | **66** |  |
| a^*^ | 1.07±0.11^a^ | 1.20±0.1^a^ | 1.17±0.20^a^ | 0.67 |
| Colorscore | 3.09±0.04^a^ | 3.02±0.04^a^ | 3.07±0.08^a^ | 0.48 |
| Marbling | 3.22±0.06^a^ | 3.14±0.06^a^ | 3.20±0.12^a^ | 0.67 |
| **DYL (N = 546)** |  |  |  |  |
| **N** | **434** | **111** | **1** |  |
| a^*^ | 1.20±0.06^a^ | 1.17±0.09^a^ | 0.76 | 0.37 |
| Colorscore | 3.02±0.03^a^ | 3.05±0.05^a^ | 3 | 0.69 |
| Marbling | 2.72±0.04^a^ | 2.70±0.06^a^ | 2 | 0.41 |
| IMF (%) | 1.82±0.03^a^ | 1.84±0.05^a^ | 0.73 | 0.3 |

Z and z represent two different nucleotide repeats: (AAC)_2_ (CAG)_2_ (TCC)_2_ and (AC)_3_ and in H1 and H2-H4, respectively (see Table1)
